# Ginseng Promotes White Adipose Tissue Browning: A Network of Thermogenic Pathways and Gut Microbiota Modulation

**DOI:** 10.3390/foods15061037

**Published:** 2026-03-16

**Authors:** Luran Yang, Yueqiao Li, Jinghui Wang, Da Li, Yuguang He, Xinyu Miao, Mubai Sun, Honghong Niu, Zhengyang Luo, Mei Hua, Xinyan Zhou

**Affiliations:** 1 Institute of Agro-Food Technology, Jilin Academy of Agricultural Science (Northeast Agricultural Research Center of China), Changchun 130033, Chinawangjhjaas@163.com (J.W.);; 2 Jinhua Academy of Zhejiang Chinese Medical University, Jinhua 321000, China; 3 College of Environment and Resources, College of Carbon Neutrality, Zhejiang A & F University, Hangzhou 311300, China

**Keywords:** ginseng, white adipose tissue browning, thermogenesis, gut microbiota, energy metabolism, gut-organ axis

## Abstract

Obesity is characterized by abnormal adipose tissue expansion and energy metabolism imbalance. Browning of white adipose tissue (WAT), wherein white adipocytes acquire thermogenic properties similar to brown adipose tissue, represents a key mechanism for increasing energy expenditure. Although ginseng (*Panax ginseng* C.A. Meyer) is widely recognized as a health-promoting botanical, its role in WAT browning has not been fully elucidated. This review summarizes evidence that ginseng and its bioactive components regulate major thermogenic pathways, including β-adrenergic/cyclic adenosine monophosphate-protein kinase (cAMP-PKA) signaling, AMP-activated protein kinase (AMPK), and the peroxisome proliferator-activated receptor γ (PPARγ)/coactivator 1α (PGC-1α) axis, thereby upregulating key markers such as uncoupling protein 1 (UCP1), PR domain containing 16 (PRDM16) and type II iodothyronine deiodinase (DIO2). These effects promote mitochondrial function and fatty acid oxidation, reduce lipogenesis, alleviate inflammation, and improve insulin sensitivity, collectively fostering a microenvironment conducive to browning. Furthermore, fermentation has been found to enhance the bioactivity and thermogenic efficacy of ginseng. Recent evidence indicates that gut microbiota and their metabolites—such as short-chain fatty acids, unsaturated fatty acids, and bile acids—play a notable role in ginseng-induced thermogenesis via receptors including G-protein-coupled receptor 41/43 (GPR41/43), takeda G-protein-coupled receptor 5 (TGR5), and farnesoid X receptor (FXR). These multi-organ interaction networks involving the gut–fat, gut–liver, and gut–brain axes reflect the role of ginseng in integrating systemic metabolism. In summary, this review discusses the multi-level regulatory network through which ginseng promotes WAT browning, providing a mechanistic basis for its potential application in body weight and metabolic health management.

## 1. Introduction

Over recent decades, profound shifts in global lifestyle and dietary patterns have been accompanied by a steady rise in the prevalence of overweight and obesity, which has emerged as a major global public health challenge [1]. According to the *World Obesity Atlas 2024* published by the World Obesity Federation, approximately 42% of adults worldwide were affected by overweight or obesity in 2020, and this proportion is expected to increase to 54% by 2035 [2]. Obesity is characterized by excessive adipose tissue accumulation and disruption of energy balance, and it constitutes a major risk factor for a range of chronic metabolic disorders, including insulin resistance, type 2 diabetes mellitus, cardiovascular diseases, and non-alcoholic fatty liver disease [3,4]. Given its complex etiology and long-term health consequences, there remains a pressing need to further elucidate the molecular mechanisms underlying obesity development and to identify safe, effective, and sustainable intervention strategies of both scientific and clinical relevance.

Mammalian adipose tissue is generally classified into white adipose tissue (WAT) and brown adipose tissue (BAT) based on differences in cellular morphology, anatomical distribution, and functional characteristics [5]. WAT primarily serves as the main site for energy storage, whereas BAT is specialized for energy dissipation via non-shivering thermogenesis, a process largely mediated by uncoupling protein 1 (UCP1) located in the inner mitochondrial membrane [6]. Under physiological or environmental stimuli such as chronic cold exposure or sustained activation of β-adrenergic signaling, WAT displays marked phenotypic plasticity and can undergo a browning process, leading to the emergence of beige adipocytes with BAT-like thermogenic properties. This adaptive conversion is commonly referred to as WAT browning [7,8]. The browning process is characterized by enhanced mitochondrial biogenesis and increased expression of thermogenesis-related genes, including UCP1 and type II iodothyronine deiodinase (DIO2), which together promote intracellular thyroid hormone activation and heat production [9,10]. In addition to peripheral regulatory mechanisms, accumulating evidence suggests that central factors, such as brain-derived neurotrophic factor (BDNF), may participate in the neuroendocrine regulation of WAT browning, thereby contributing to increased energy expenditure and improved metabolic capacity of adipose tissue.

WAT browning is a highly complex biological process that is coordinately regulated by multiple mechanisms, including neuroendocrine signaling, cellular energy-sensing pathways, and nutritional metabolic cues [11]. According to the classical regulatory model, PR domain-containing protein 16 (PRDM16) and sirtuin 1 (SIRT1) act in concert with sympathetic nervous system activation to mediate β_3_-adrenergic receptor signaling, thereby modulating the transcriptional activity of peroxisome proliferator-activated receptor γ (PPARγ). This regulatory cascade relies on the induction and nuclear translocation of the transcriptional coactivator 1α (PGC-1α), as well as its interaction with the promoter regions of thermogenic genes [12,13,14]. In parallel, AMP-activated protein kinase (AMPK), a central sensor of cellular energy status, facilitates WAT browning by suppressing lipogenic pathways while enhancing fatty acid oxidation and mitochondrial function [15,16]. Collectively, these coordinated signaling events highlight the induction of beige adipocyte formation as a promising strategy to increase energy expenditure and ameliorate obesity-associated metabolic disorders [17].

**Figure 1:**
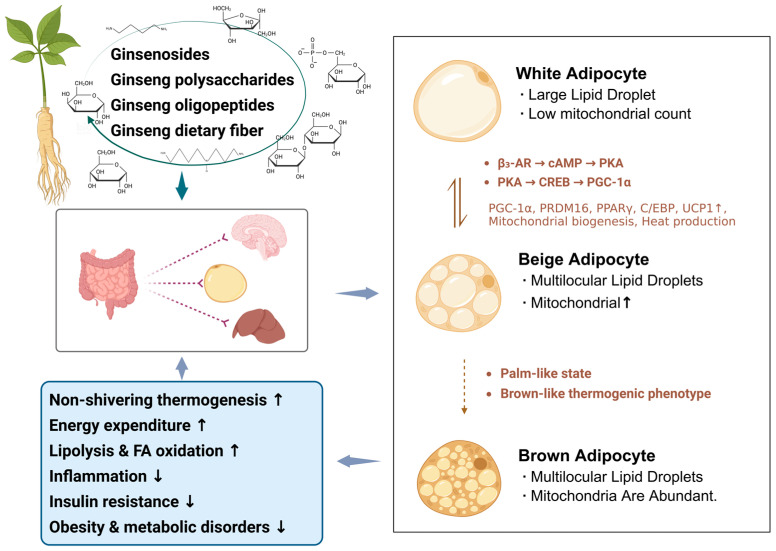
Regulatory effects of ginseng on white adipose tissue browning and energy metabolism. The solid arrows represent direct regulatory pathways by ginseng constituents, while dashed arrows indicate indirect metabolic influences (BioRender.com., Agreement number: RA29BAROME). Upward (↑) and downward (↓) arrows denote increases and decreases in molecular markers or metabolic functions. The bidirectional arrow (⇌) symbolizes the phenotypic transition between white, beige, and brown adipocytes.

At present, a variety of strategies have been explored to activate brown adipose tissue and promote WAT browning [18,19]. However, many of these approaches depend on strong pharmacological or physiological stimulation and may be associated with potential adverse effects on the cardiovascular and nervous systems, thereby limiting their suitability for long-term application [20]. In this context, increasing attention has shifted toward naturally derived interventions that are generally considered safer and exert relatively moderate biological effects. *Panax ginseng* C.A. Meyer (ginseng), a representative medicinal and edible homologous plant, has been extensively investigated and reported to exert beneficial effects on body weight regulation, insulin sensitivity, and glucose and lipid metabolism. These properties suggest that ginseng may simultaneously promote WAT browning and enhance brown adipose tissue thermogenic capacity [21,22,23]. Existing studies indicate that ginseng is rich in ginsenosides, polysaccharides, and other bioactive constituents, which are regarded as the primary material basis underlying its metabolic regulatory activities [24]. These components have been shown to activate key signaling pathways, including protein kinase A (PKA) and AMPK, thereby promoting lipolysis and fatty acid oxidation while suppressing inflammatory responses and alleviating hepatic injury [25,26]. Nevertheless, ginseng also contains fatty acids and other minor constituents with distinct biological activities, and the precise molecular mechanisms through which these components contribute to adipose tissue browning remain incompletely understood. Accordingly, this review focuses on the major bioactive components of ginseng, including ginsenosides, polysaccharides, oligosaccharides, and dietary fiber, and summarizes their roles in promoting adipose tissue browning and improving metabolic homeostasis. In addition, the signaling pathways and gut–organ interactions through which ginseng modulates the functional interplay between white and brown adipose tissues are discussed (Figure 1), with the aim of providing a theoretical basis for the development of ginseng-derived functional ingredients and nutritional intervention strategies.

## 2. Molecular Basis of Ginseng in White Adipose Tissue Browning

Intracellular signaling pathways involved in WAT browning have been widely investigated in metabolic research. Accumulating evidence indicates that pathways related to sympathetic stimulation, cellular energy sensing, and transcriptional regulation contribute to the modulation of thermogenic activity. Among these regulatory factors, the major ginseng-derived bioactive components implicated in adipose tissue browning include ginsenosides, polysaccharides, oligosaccharides, and dietary fiber. Under the influence of these components, adipocytes undergo coordinated metabolic reprogramming, characterized by enhanced energy substrate utilization, increased mitochondrial activity, and activation of thermogenesis-related signaling pathways. Importantly, these pathways function in an interconnected rather than isolated manner, converging on key molecular events such as transcription factor activation, modulation of mitochondrial biogenesis, and maintenance of systemic energy homeostasis.

**Figure 2:**
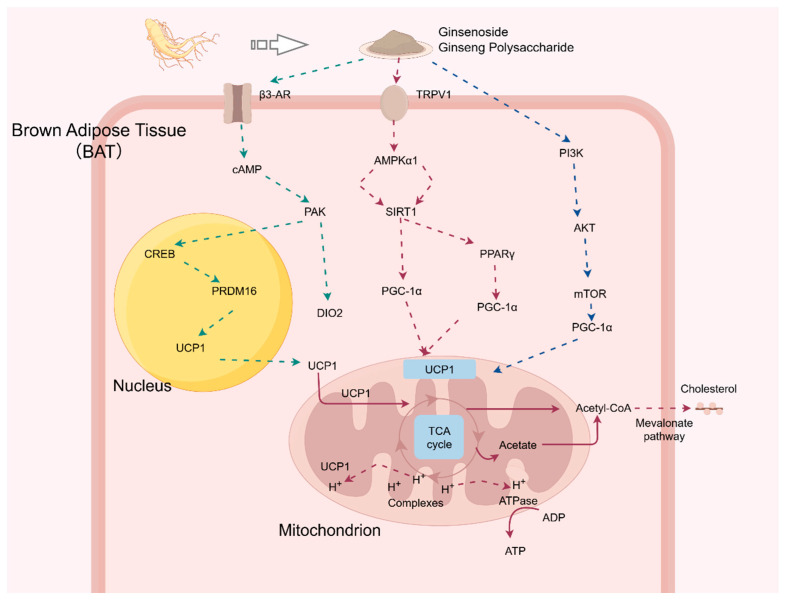
Ginseng-derived bioactive components promote white adipose tissue browning through multiple signaling pathways (Figdraw.com., Agreement number: SITOP69a4f). The pathways are color-coded: green arrows for the β3-AR signaling cascade, red arrows for the AMPK/SIRT1-mediated energy-sensing axis, and blue arrows for the PI3K/AKT/mTOR pathway. Arrows pointing to the nucleus signify transcriptional activation, while those directed at mitochondria illustrate the regulation of fatty acid oxidation and thermogenesis.

These signaling pathways and their upstream regulatory inputs provide a mechanistic basis for understanding how ginseng-derived components may influence adipocyte metabolic regulation. The principal signaling pathways, key molecular targets, and their interactions involved in this regulatory process are schematically summarized in Figure 2.

### 2.1. Ginsenosides and Thermogenic Signaling in Adipocytes

Ginsenosides are among the most important bioactive constituents of ginseng. Based on their relative abundance in the plant and structural characteristics, ginsenosides are commonly classified into major and rare types [27]. Major ginsenosides, including Rb1, Rb2, Rc, Rd, Re, and Rg1, are present at relatively high levels in both fresh and dried ginseng; however, owing to their multiple glycosyl moieties, they generally exhibit limited bioavailability. In contrast, rare ginsenosides—such as Rg3, Rh2, Rk1, Rk2, aPPD, and aPPT—are mainly produced through biotransformation processes and contain fewer sugar residues. These structural differences have been associated with differences in bioavailability and biological activity in vivo [28,29].

#### 2.1.1. Major Ginsenosides and β_3_-AR–Related Signaling

Previous studies report that major ginsenosides are associated with modulation of intracellular signaling pathways related to energy metabolism [30]. Among these compounds, ginsenoside Rb1 has been the most extensively investigated representative. Emerging evidence from in vitro adipocyte models and diet-induced obese mouse studies suggests that Rb1 treatment may correlate with increased AMPKα phosphorylation and shifts in lipolytic marker expression [31,32]. Further studies have reported that ginsenoside Rb1 modulates AMPK signaling in both 3T3-L1 adipocytes and high-fat diet-induced mouse models [33]. In these systems, Rb1 treatment was associated with increased expression of browning-related markers and features consistent with enhanced thermogenic capacity. This response is characterized by downregulation of adipogenic markers such as CCAAT/enhancer-binding protein alpha (C/EBPα) and sterol regulatory element-binding protein-1c (SREBP-1c), accompanied by upregulation of thermogenic genes including PPARγ, PRDM16, PGC-1α, and UCP1. In addition, Rb1 has been reported to regulate hepatic glucose production via AMPK-dependent mechanisms, alleviate hepatic lipid accumulation, and suppress inflammatory signaling pathways such as nuclear factor-kappa B (NF-κB) and NLR family pyrin domain containing protein 3 (NLRP3) inflammasome activation, thereby providing metabolic support for adipose tissue browning and the maintenance of systemic energy homeostasis [34,35]. While these data point to the potential involvement of β3-adrenergic receptor (AR) signaling and broader energy-sensing networks, definitive validation at the receptor level remains limited. Moreover, in most current experimental models, the causal linkage between AMPK activation and the reduction in lipid accumulation has not yet been fully established.

Ginsenoside Rb2 has also been reported to participate in metabolic regulation associated with adipose tissue browning, with its mechanism of action potentially involving modulation of mammalian target of rapamycin (mTOR) activity via the AMPK-Unc-51-like kinase 1 (ULK1) signaling axis [36]. Available studies indicate that Rb2 intervention influences the expression of autophagy-related genes, including Atg5, Atg7, and Beclin-1, and is accompanied by increased expression of brown adipose tissue-associated marker genes such as UCP1, PGC-1α, DIO2, and nuclear respiratory factor 1 (NRF-1) [37]. In addition, Rb2 has been shown to enhance protein kinase B (AKT) phosphorylation at the Ser473 site through regulation of the phosphoinositide 3-kinase (PI3K)/AKT signaling pathway, a response associated with improved BAT function and the emergence of WAT browning phenotype [38].

Beyond Rb-type ginsenosides, other compounds such as ginsenosides Rg1 and Rd have also been reported to exert regulatory effects on adipose tissue browning. Ginsenoside Rg1 has been reported to increase AMPK phosphorylation through the insulin receptor substrate 1 (IRS-1)/PI3K/Akt pathway and inhibit acetyl-CoA carboxylase (ACC) activity, thereby promoting fatty acid oxidation and enhancing energy expenditure [39]. Moreover, Rg1 has been shown to downregulate the expression of lipogenesis-related transcription factors, including PPARγ, C/EBP, and SREBP-1c, further supporting metabolic processes associated with adipose tissue browning [40]. By contrast, ginsenoside Rd appears to act primarily through the cAMP/PKA signaling pathway, enhancing cAMP response element-binding protein (CREB) activity and promoting UCP1 transcription. Consistent browning-related effects of Rd have been observed in both brown adipose tissue from obese mouse models and in vitro-cultured adipocytes [41,42].

#### 2.1.2. Rare Ginsenosides and AMPK-Related Signaling

Rare ginsenosides are generally considered to exhibit higher biological activity than their major counterparts and have shown favorable effects in studies related to lipid lowering and metabolic regulation [43]. Among these compounds, ginsenoside F1 has been reported to interact with the β_3_-AR and effectively activate the cAMP/PKA/CREB signaling cascade, leading to increased expression of thermogenic markers such as UCP1, PRDM16, and PGC-1α, together with enhanced thermogenic capacity and energy expenditure [44]. Notably, the browning-related effects of ginsenoside F1 appear to be largely confined to WAT, whereas comparatively weaker effects have been observed in BAT and hepatic UCP1 expression. This tissue-selective response suggests that F1 may preferentially modulate WAT browning rather than directly activating classical BAT thermogenesis [45].

In addition to F1, rare ginsenosides such as Rg1 and Rg3 have also been implicated in the regulation of adipose tissue browning, primarily through AMPK-related signaling pathways. Ginsenoside Rg1 has been shown to modulate AMPK and PPARγ signaling, thereby promoting the expression of thermogenesis-associated genes and mitochondrial function-related genes, while simultaneously suppressing the expression of lipogenesis-related transcription factors. These coordinated regulatory effects are closely associated with the induction of a WAT browning phenotype [46]. By contrast, in diet-induced obese mouse models, Rg3 administration has been linked to enhanced AMPK phosphorylation, coupled with the upregulation of key thermogenic markers, including PGC-1α and PRDM16. Concurrently, the observed downregulation of transcriptional activity related to mTORC1, PPARγ, and C/EBPα suggests a potential mechanism of coordinated metabolic reprogramming. However, it remains to be elucidated whether AMPK activation acts as a primary initiator of these metabolic shifts or as a secondary adaptive response to Rg3 intervention [47,48,49].

Although a number of ginsenosides have been reported to promote white adipose tissue browning, their effects are not consistent across experimental settings. The mechanisms described in this section mainly concern the direct effects of ginseng and its constituents on adipocyte signaling. Rb1 and Rb2, for instance, have shown relatively stable activation of AMPK-related pathways in both 3T3-L1 adipocytes and high-fat diet-induced obese mice. In contrast, the browning effects of Rg1 or Rd appear to depend more strongly on experimental context and are sometimes confined to in vitro systems. Similarly, Rg3 has been shown to enhance mitochondrial biogenesis and UCP1 expression in obese mouse models, whereas its effects are less evident under normal metabolic conditions. Rare ginsenosides such as F1 have demonstrated preferential induction of browning markers in white adipose tissue, with comparatively limited activation of classical brown adipose depots, suggesting potential tissue specificity. These findings indicate that browning responses vary according to ginsenoside structure, adipose depot type, and metabolic background. Direct comparison between studies is therefore difficult, and caution is needed when generalizing the browning capacity of individual ginsenosides.

### 2.2. Non-Saponin Constituents and Metabolic Regulation

In addition to ginsenosides, ginseng contains several non-saponin constituents, including polysaccharides, oligopeptides, and dietary fiber. These components have been studied primarily for their metabolic and anti-inflammatory effects. Compared with ginsenosides, which have been directly examined in adipocyte models, the evidence linking non-saponin fractions to WAT browning is less direct. Most studies report changes in metabolic parameters rather than direct evaluation of thermogenic activation.

Ginseng polysaccharides are structurally diverse macromolecules composed of neutral and acidic fractions, as well as pectic polysaccharides enriched in rhamnose, galactose, and arabinose residues [50]. Experimental work has largely focused on their effects in diet-induced obese mouse models, where improvements in lipid metabolism, insulin sensitivity, and inflammatory markers have been observed [51,52]. At the cellular level, some polysaccharide fractions have been associated with modulation of nutrient-sensing pathways, including mTOR-related signaling, and with reduced lipid synthesis under metabolic stress. These findings suggest an influence on adipocyte metabolic regulation. Direct evidence demonstrating sustained induction of canonical thermogenic markers such as UCP1 or PRDM16, however, remains limited. In most cases, conclusions regarding browning are inferred from improved metabolic status rather than from direct functional measurements. Overall, current data support a role for ginseng polysaccharides in shaping the metabolic milieu, while direct activation of adipocyte thermogenic programs has not been firmly established.

Ginseng oligopeptides, characterized by a relatively low molecular weight and higher bioavailability, have mainly been investigated in the context of metabolic regulation. Reported effects include modulation of LPS–TLR4–NF-κB, IRS-1/PI3K/Akt, and AMPK signaling pathways [28,29,30]. Associations with the NAD^+^/SIRT1/PGC-1α axis have also been described, suggesting possible effects on mitochondrial function and oxidative metabolism. These pathway-level observations indicate adjustments in cellular energy handling. Direct experimental evidence demonstrating beige adipocyte differentiation or sustained thermogenic activation is currently sparse. In many studies, thermogenic implications are derived from signaling changes rather than validated through measurements of oxygen consumption or heat production. At present, the relationship between ginseng oligopeptides and WAT browning should therefore be considered indirect.

Ginseng dietary fiber has been examined primarily for its effects on glucose homeostasis, lipid metabolism, and gut microbiota composition [53,54]. Because dietary fiber is minimally absorbed, its biological effects are largely mediated through microbial fermentation and downstream systemic signaling. Although improvements in metabolic parameters have been reported, direct evidence of adipocyte-specific thermogenic activation following dietary fiber intervention is lacking. Interpretations suggesting a role in browning are generally based on indirect metabolic indicators rather than on direct assessment of thermogenic gene expression or function. Current findings are therefore more consistent with systemic metabolic remodeling than with confirmed induction of classical browning pathways.

**Figure 3:**
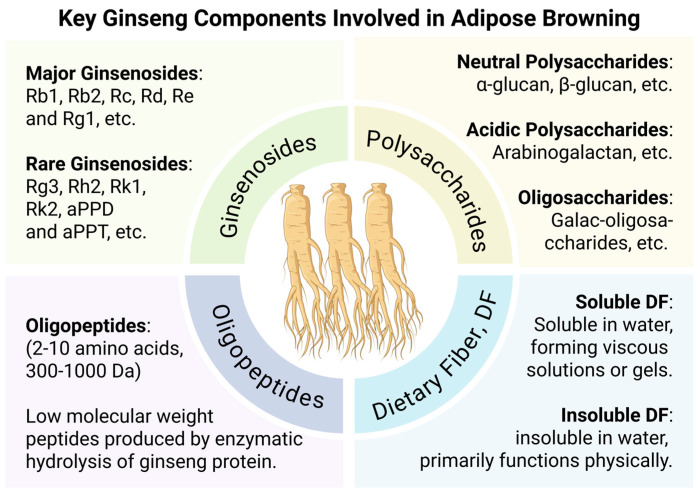
Classification of major ginseng-derived bioactive components associated with white adipose tissue browning (BioRender.com. Agreement number: FP29BE8UTT).

Available evidence at the cellular and molecular levels suggests that ginseng and its bioactive constituents are associated with the regulation of WAT browning. Different classes of ginseng-derived components—including ginsenosides, polysaccharides, oligopeptides, and dietary fiber—exhibit distinct structural characteristics, bioavailability profiles, and metabolic properties. Experimental studies indicate that these components influence cellular energy metabolism, mitochondrial function, and the expression of thermogenesis-related genes in adipocytes. An overview of the major categories of ginseng bioactive components and their current research focus in relation to adipose tissue browning is presented in Figure 3.

### 2.3. Interpreting Molecular Changes and Thermogenic Outcomes

The findings summarized in the preceding sections indicate that ginseng intervention is associated with modulation of molecular pathways involved in energy metabolism and adipocyte function [16,19,20,31,33,42]. In several experimental models, these signaling alterations are accompanied by increased expression of thermogenesis-related genes. However, pathway activation and transcriptional changes do not necessarily correspond to sustained thermogenic function. Energy-sensing pathways such as AMPK- and PPARγ-related signaling are central regulators of cellular metabolism and are activated under a variety of metabolic conditions. Their activation in adipocytes is therefore not specific to beige differentiation. Similarly, increased expression of markers including UCP1, PRDM16, or PGC-1α reflects transcriptional remodeling but does not by itself demonstrate increased oxidative flux or heat production. In many studies, molecular analyses are not consistently paired with direct functional measurements, such as indirect calorimetry or oxygen consumption assays. As a result, the relationship between gene expression changes and thermogenic output remains incompletely defined.

Some molecular responses described in the literature may also represent metabolic adaptation rather than stable phenotypic conversion. Alterations in mitochondrial activity and substrate utilization frequently occur in response to changes in energy availability or metabolic stress. Under such conditions, upregulation of thermogenesis-associated genes may reflect broader adjustments in metabolic capacity rather than selective activation of a dedicated browning program. Distinguishing adaptive metabolic remodeling from sustained beige adipocyte differentiation requires integrated functional and longitudinal assessment. Interpretation is further influenced by differences in experimental design. Most mechanistic data are derived from in vitro adipocyte systems or diet-induced obese animal models [55]. While these models allow detailed analysis of intracellular signaling, they do not fully capture systemic regulation. Outcomes may vary according to genetic background, adipose depot, dietary composition, metabolic status, and duration of treatment [55].

In addition, purified compounds are often administered at concentrations that exceed nutritionally achievable exposure levels. Because metabolic signaling frequently displays dose- and context-dependent behavior [56,57,58], variations in exposure conditions may affect both the magnitude and reproducibility of reported effects. The absence of standardized dosing strategies and consistent functional endpoints further limits comparison across studies. Adipose tissue function is regulated within a broader metabolic environment. Following oral intake, ginseng-derived compounds undergo digestion and metabolic transformation before reaching peripheral tissues. Consequently, molecular changes observed in adipocytes may reflect indirect systemic influences rather than direct receptor-level activation. Consideration of these systemic factors is important for interpreting thermogenic outcomes in a physiologically relevant context. Overall, available data indicate that modulation of signaling pathways and thermogenesis-related gene expression reflects altered cellular energy regulation. Whether these changes result in sustained thermogenic remodeling remains to be established through comprehensive functional evaluation under conditions that approximate realistic exposure.

## 3. The Role of the Intestinal Microbiota in the Process of Ginseng Promoting the Browning of White Adipose Tissue

Most existing studies have concentrated primarily on adipose tissue itself, while comparatively less attention has been paid to systemic metabolic regulation following dietary ginseng intake. Because ginseng-derived compounds are consumed orally, their digestion, absorption, and metabolic transformation occur largely within the gastrointestinal tract before reaching peripheral tissues. In this context, gut microbiota and related metabolites may influence whole-body energy homeostasis and indirectly affect adipose tissue function. Further investigation of these systemic interactions may help clarify how ginseng intake contributes to adipose tissue remodeling within the broader framework of energy metabolism.

Recent research has confirmed that ginseng may influence adipose tissue function indirectly by modulating gut microbiota composition and metabolite production. These changes are characterized by an increased abundance of potentially beneficial bacterial taxa, including *Bacteroides*, *Proteobacteria*, *Verrucomicrobia*, *Bifidobacterium*, *Parabacteroides*, and *Akkermansia*, while the relative abundance of bacterial groups associated with inflammation or metabolic disturbance, such as *Deferribacteres* and *Helicobacter*, is reduced. It should be noted that the effects of ginseng on certain bacterial genera, including *Lactobacillus*, may vary depending on specific strains and experimental conditions. Detailed alterations in gut microbiota composition are summarized in Table 1. Accompanied by these microbiota shifts, ginseng intake has been associated with changes in gut microbiota-derived metabolites, including short-chain fatty acids, medium- and long-chain fatty acids, and bile acids. These metabolites are known to act as key signaling molecules that participate in adipose tissue remodeling and energy metabolism, and they may exert regulatory effects during the process of WAT browning.

### 3.1. Ginseng Participates in White Adipose Tissue Browning Through Modulation of Short-Chain Fatty Acid Metabolism

Evidence from cellular and animal models indicates that short-chain fatty acid (SCFA) supplementation correlates with the upregulation of canonical thermogenic markers and the activation of AMPK-dependent metabolic pathways. Mechanistically, these observations appear to be mediated, at least in part, by G-protein-coupled receptor GPR41/43 signaling—a family of G-protein-coupled receptors—alongside epigenetic modulation via histone deacetylase (HDAC) inhibition [59,60,61,62,63]. Acetate, propionate, and butyrate, in particular, have been shown to activate these receptors and may facilitate cellular uptake via monocarboxylate transporter 1 (MCT1) [64,65]. Such metabolic flux frequently correlates with the expression of key thermogenic regulators, including PRDM16 and UCP1, suggesting that SCFAs primarily engage established energy-sensing networks, such as the AMPK-PGC-1α signaling axis, rather than functioning through independent or novel pathways [60,61].

Beyond these direct cellular effects, SCFAs appear to exert broader systemic metabolic control by stimulating enteroendocrine cells to secrete gut hormones, including glucagon-like peptide-1 (GLP-1) and peptide YY (PYY) [61,66]. These hormones contribute to the regulation of energy intake and the maintenance of systemic homeostasis, thereby providing essential endocrine support for browning-associated metabolic processes in adipose tissue. While these factors suggest that SCFAs serve as functional mediators within the “gut microbiota–metabolite–adipose tissue” axis, the interplay between these circulating hormones and local adipose tissue remodeling remains a complex and multifaceted field of study.

Collectively, these findings position SCFAs as potential regulators of energy metabolism; however, direct causal evidence linking this axis specifically to ginseng-induced metabolic remodeling remains insufficient. Given that ginseng components are known to modulate gut microbiota composition, it is hypothesized that this herbal intervention may exert synergistic regulatory effects on SCFA-driven WAT browning. Nevertheless, the precise sequence of these microbial-metabolic events, as well as the relative contribution of systemic versus local signaling, requires further rigorous investigation to delineate the clinical relevance of these pathways in human metabolic health.

### 3.2. Ginseng Participates in White Adipose Tissue Browning Through Modulation of Medium- and Long-Chain Fatty Acid Metabolism

Medium-chain fatty acids (MCFAs) and certain unsaturated long-chain fatty acids function not only as efficient energy substrates but also as signaling molecules, playing important roles in promoting adipose tissue browning and increasing energy expenditure. Although direct evidence for large-scale MCFA production by specific gut microbial taxa remains limited, accumulating studies indicate that MCFAs are rapidly absorbed and preferentially oxidized. These metabolic characteristics contribute to elevated energy expenditure and may facilitate the transition of adipose tissue toward a thermogenic phenotype [67]. Consistent with this notion, mixed medium- and long-chain triacylglycerols (MLCTs) containing defined proportions of MCFAs have been reported to reduce body weight and improve overall metabolic status. These effects are associated with upregulation of hepatic genes involved in fatty acid β-oxidation, such as PPARα and long-chain acyl-CoA dehydrogenase (LCAD), together with suppression of lipogenesis-related genes, including fatty acid synthase (FAS), ACC1, and SREBP-1c [68]. Collectively, these findings suggest that modulation of medium- and long-chain fatty acid metabolism may represent an additional pathway through which ginseng contributes to adipose tissue browning and systemic metabolic regulation.

Recent studies further indicate that ginseng extracts can reshape gut microbiota composition and increase the relative abundance of *Enterococcus faecalis* in obese mouse models. This bacterial species has been reported to possess the capacity to synthesize the unsaturated long-chain fatty acid myristoleic acid (MA) [69]. As a bioactive lipid mediator, MA has been implicated in the activation of β_3_-AR-related signaling and has been associated with increased expression of UCP1, enhanced BAT function, and the induction of a WAT browning phenotype.

Additional support for this mechanism is provided by antibiotic intervention and fecal microbiota transplantation studies, which suggest that ginseng may exert its regulatory effects on body weight and energy metabolism through the “gut microbiota–fatty acid metabolite” axis. Notably, genetic disruption of acyl-CoA thioesterase (ACOT), a key enzyme involved in MA biosynthesis, markedly attenuated these metabolic benefits, thereby supporting a critical mediating role for MA in this regulatory process [70,71]. Taken together, these findings suggest that ginseng, by remodeling gut microbiota structure and associated fatty acid metabolic profiles, may exert synergistic effects on adipose tissue thermogenic regulation and contribute to the amelioration of obesity-related metabolic disturbances.

### 3.3. Ginseng Participates in White Adipose Tissue Browning Through Modulation of Bile Acid Metabolism

Bile acids serve not only as essential mediators of dietary lipid absorption but also as important signaling molecules involved in the regulation of energy metabolism and adipose tissue function. Previous studies have demonstrated that ginseng and its ginsenoside constituents can modulate bile acid metabolism by regulating the farnesoid X receptor (FXR)/cholesterol 7α-hydroxylase (CYP7A1)-related pathway, thereby influencing enterohepatic bile acid circulation and reshaping bile acid pool composition in association with lipid metabolic regulation [72,73]. Among these components, ginsenoside Rg1 has been reported to alter fecal bile acid profiles in obese mouse models, characterized by increased levels of cholic acid (CA) and its conjugated forms, such as taurocholic acid (TCA), together with reduced levels of certain glycine-conjugated bile acids. These changes suggest that Rg1 may contribute to the maintenance of metabolic homeostasis through modulation of bile acid composition [74]. In addition, ginsenoside Rh4 has been shown to increase the relative abundance of the mucin-degrading bacterium *Akkermansia muciniphila*, enhance the activity of 7α-hydroxysteroid dehydrogenase (7α-HSDH), and promote the conversion of primary bile acids into secondary bile acids. Through these microbiota-mediated mechanisms, Rh4 may indirectly influence adipose tissue function and systemic energy metabolism at the whole-body level [63,75].

At the molecular level, bile acids regulate adipose tissue energy metabolism primarily through activation of the membrane receptor Takeda G protein–coupled receptor 5 (TGR5/GPBAR1) and the nuclear receptor FXR. TGR5 is preferentially activated by secondary bile acids, such as deoxycholic acid (DCA) and its conjugated forms. Activation of this pathway has been associated with enhanced thermogenic capacity of BAT, induction of a WAT browning phenotype, and increased secretion of GLP-1 from enteroendocrine cells, collectively supporting elevated energy expenditure [76,77]. In parallel, FXR plays a central role in lipid metabolic regulation by modulating signaling pathways involving PPARα and SIRT1, thereby promoting fatty acid oxidation while limiting lipid accumulation. Through these coordinated actions, FXR and TGR5 constitute a complementary bile acid-responsive regulatory network that contributes to the maintenance of systemic energy homeostasis [78,79]. Taken together, these findings suggest that ginseng, by modulating bile acid metabolism and bile acid-associated signaling pathways, may exert regulatory effects within the “bile acid–receptor–adipose tissue” axis. This mode of action may provide system-level support for adipose tissue browning-related metabolic processes and contribute to the improvement of obesity-associated metabolic disturbances.

## 4. Ginseng May Promotes White Adipose Tissue Browning via the “Gut–X” Organ Axis

In recent years, advances in gut microbiome research have underscored the pivotal role of the gut microbiota in the development and progression of obesity. The microbiota forms a complex, multi-organ interactive network—the “gut–X” Organ Axis—through which bidirectional communication occurs between the gastrointestinal tract and host organs, including the central nervous system, liver, and adipose tissue [80,81]. Through this integrated network, the gut microbiota contributes to the coordinated regulation of energy metabolism, appetite-related behaviors, and body weight homeostasis.

More and more evidence suggests that ginseng may indirectly create a “permissive” metabolic environment through the systemic network constructed by the gut microbiota. This dual-action framework—distinguishing between direct intracellular signaling and systemic modulation—is summarized in Table 2. From a systems-level perspective, ginseng may influence thermogenesis-related metabolic processes by modulating gut microbiota composition and the associated metabolite profile. This engages multiple interconnected regulatory axes—such as the gut–adipose, gut–liver, and gut–brain axes—thereby transforming ginseng from a simple molecular activator into a coordinator of systemic metabolic remodeling.

### 4.1. Ginseng Participates in the Regulation of White Adipose Tissue Browning Through the Gut–Adipose Axis

By contrast, the mechanisms outlined below relate to indirect influences on adipose tissue browning, driven by microbiota-derived signals and immune processes rather than direct activation of adipocyte pathways. The gut–adipose axis (GAA) contributes to the regulation of adipose tissue browning through multiple interconnected mechanisms, including modulation of gut microbiota composition and their metabolites, short-chain fatty acid-mediated signaling, gut hormone secretion, and immune–neural interactions [82,83,84]. Among these regulatory components, the gut microbiota is widely regarded as a central hub that links dietary cues to adipose tissue function. Accumulating evidence indicates that specific gut microbial communities can promote the production of type 2 immune-associated cytokines, such as interleukin (IL)-4, IL-13, and IL-5, thereby driving the polarization of anti-inflammatory M2 macrophages. This immune remodeling improves the local immune microenvironment of adipose tissue and is closely associated with brown adipocyte formation and enhanced thermogenic capacity [85,86]. Accordingly, immune-mediated regulation along the gut–adipose axis is considered a key factor for the initiation and maintenance of adipose tissue browning.

In germ-free animal models or under conditions of gut microbiota remodeling, the emergence of adipose tissue browning phenotypes is frequently accompanied by a marked upregulation of angiopoietin-like protein 4 (ANGPTL4), together with increased expression of PGC-1α and genes involved in fatty acid oxidation [87,88]. As a key mediator of gut microbiota–host interactions, ANGPTL4 regulates lipid uptake and fatty acid utilization, thereby shaping the metabolic characteristics of adipose tissue. These observations suggest that alterations in gut microbiota can reprogram lipid metabolic signaling networks and exert systemic effects on adipose tissue phenotype and thermogenic potential.

**Figure 4:**
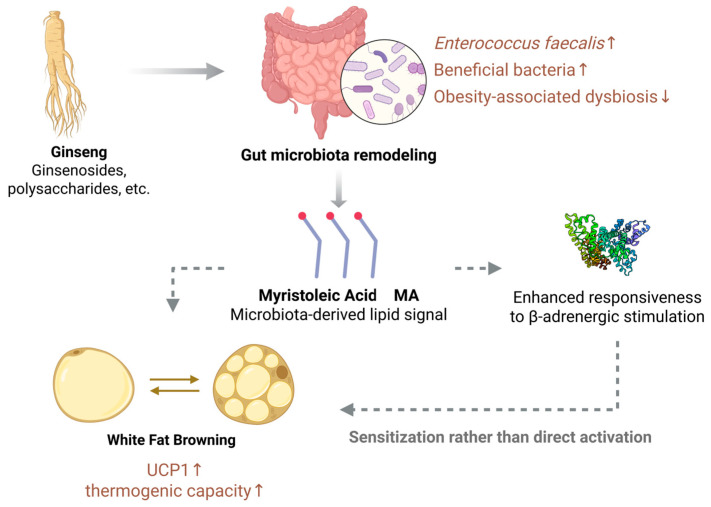
Potential mechanisms by which ginseng promotes adipose tissue browning through the gut–adipose axis (BioRender.com. Agreement number: KT29BE7WRF). The arrow from ginseng to the gut microbiota denotes the modulation of microbial composition. The production of myristoleic acid (MA) is shown as a microbial-derived metabolite. Dashed arrows indicate that MA functions as a sensitizer to β-adrenergic stimulation, rather than a direct agonist, to facilitate the browning process.

Within this context, research into ginseng-induced metabolic shifts offers insights into the gut–adipose axis as a potential mediator of thermogenic regulation. Studies in obese mouse models have indicated that ginseng administration correlates with an increased relative abundance of *Enterococcus faecalis* in the gut, a shift that appears to coincide with elevated systemic concentrations of MA—an unsaturated long-chain fatty acid. As a putative bioactive lipid mediator, MA has been implicated in β_3_-AR-related signaling. Preliminary findings associate MA levels with increased UCP1 expression, enhanced BAT thermogenic function, and the induction of a WAT browning phenotype [89,90], as conceptually illustrated in Figure 4. These observations suggest that ginseng-mediated remodeling of the gut microbiota may be linked to enhanced UCP1 expression and augmented BAT activity through changes in the circulating lipid profile. However, while data suggest that MA might modulate β_3_-AR pathways to facilitate thermogenic responses, direct mechanistic validation—including high-affinity receptor-binding assays or targeted loss- and gain-of-function interventions—is currently limited. Consequently, the functional linkage between MA and thermogenesis has yet to be definitively established. These associations should be interpreted with a degree of caution rather than as conclusive proof of a causal mechanism driving ginseng-associated adipose tissue browning.

Taken together, the available evidence suggests that ginseng may help establish a metabolic and immune microenvironment conducive to adipose tissue browning through coordinated regulation along the gut–adipose axis, involving modulation of gut microbiota composition, immune signaling, and lipid metabolic pathways. This regulatory pattern indicates that the contribution of ginseng to adipose tissue browning is more closely related to the remodeling of the systemic metabolic context rather than direct stimulation of adipocytes alone. Through such system-level regulation, ginseng may support energy metabolism homeostasis and thereby exert potential beneficial effects on obesity and related metabolic disturbances.

### 4.2. Ginseng Participates in Adipose Tissue Browning-Related Metabolic Regulation Through the Gut–Liver Axis

The gut–liver axis (GLA) represents a bidirectional regulatory network linking the gastrointestinal tract and the liver through portal circulation, bile acid signaling, gut-derived metabolites, and immune mediators, and it plays a central role in the maintenance of systemic energy homeostasis and metabolic regulation. Recent studies indicate that the gut–liver axis is not only involved in glucose and lipid metabolism and inflammatory control but also indirectly influences mitochondrial function and thermogenic phenotypes in adipose tissue. These effects are mediated, at least in part, through alterations in bile acid composition, SCFA availability, and hepatic endocrine signaling, thereby contributing to the regulation of WAT browning [91,92]. In this context, specific gut microbial populations have been reported to induce hepatic expression of the acute-phase protein orosomucoid-2 (Orm2), which subsequently activates the gp130/IL-23R–p38 MAPK signaling pathway. Activation of this pathway has been associated with enhanced thermogenic capacity in adipose tissue and the emergence of a WAT browning phenotype [93,94]. The discussion below centers on systemic metabolic regulation rather than direct adipocyte signaling.

Within this framework, ginseng-related studies provide emerging evidence that ginsenoside Rh4 can promote the production of HDCA by modulating gut microbiota composition and bile acid metabolism. As a bioactive bile acid-derived metabolite, HDCA has been implicated in host energy metabolism regulation at the level of the gut–liver axis. Mechanistically, HDCA has been shown to suppress intestinal farnesoid X receptor (FXR) signaling, improve gut microbial composition by enriching potentially beneficial bacterial taxa, and concurrently upregulate hepatic expression of CYP7B1, PPARα, and FXR [95]. Collectively, these findings suggest that the Rh4–HDCA axis may coordinate intestinal sensing signals with hepatic metabolic responses, thereby systemically remodeling the bile acid–nuclear receptor network involved in energy metabolism and the regulation of adipose tissue browning.

Although direct evidence demonstrating that HDCA or ginsenoside Rh4 can directly induce adipose tissue browning remains limited, accumulating studies suggest that activation of hepatic PPARα serves as a critical intermediary in systemic metabolic regulation associated with adipose tissue browning. Hepatic PPARα activation is associated with enhanced lipid oxidation and improved metabolic status. Such systemic changes may create a metabolic environment that is more permissive for thermogenic gene expression in adipose tissue [96,97,98]. These coordinated effects not only support mitochondrial function in peripheral adipose tissue but also help establish a metabolic context permissive for the conversion of WAT toward a thermogenic phenotype.

**Figure 5:**
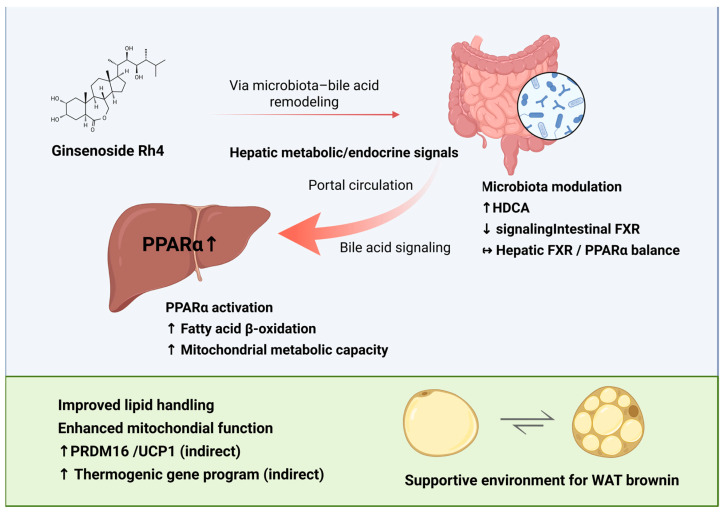
Rh4 indirectly supports adipose tissue browning through bile acid remodeling and gut-liver axis-mediated metabolic regulation (BioRender.com. Agreement number: SL29BE5VK4). The arrows connecting ginseng, the gut, and the liver depict the systemic signaling flow within the “gut-liver axis”. The arrow labeled “Portal circulation” highlights inter-organ crosstalk. The upregulation of PPARα (indicated by the upward arrow) suggests a shift in hepatic lipid metabolism that creates a permissive environment for adipose thermogenesis.

In obese mouse models, Rh4 treatment has been associated with alterations in bile acid composition and modulation of FXR-related signaling [95,97]. These changes are accompanied by enhanced hepatic PPARα activity and increased expression of lipid oxidation-associated genes, consistent with a shift toward greater fatty acid catabolism in the liver. Such findings support the view that Rh4 may influence adipose tissue function indirectly through regulation of bile acid metabolism along the intestine–liver signaling axis (Figure 5). Available evidence further indicates that Rh4 administration is associated with changes in gut microbiota composition and remodeling of the bile acid pool, including altered levels of bile acids such as HDCA [96]. Modulation of specific bile acid species may attenuate intestinal FXR signaling while favoring PPARα-driven metabolic pathways in the liver, thereby promoting systemic lipid oxidation and redistribution of metabolic substrates. Through these coordinated hepatic and intestinal adjustments, whole-body energy handling may shift toward increased oxidative utilization, which could secondarily influence adipose tissue thermogenic responsiveness. Within this framework, the effects of Rh4 on adipose tissue are best interpreted as arising from integrated metabolic regulation rather than direct adipocyte-specific activation. Elucidation of the temporal and causal relationships among bile acid remodeling, hepatic signaling, and adipose tissue responses will be necessary to clarify the precise contribution of Rh4 to thermogenic regulation.

**Table 2:** Classification of Ginseng-Mediated WAT Browning: Evidence Strength, Mechanism Type, and Perspectives on Adaptive Stress Responses.

Category	Experimental Models	Mode of Action	Regulatory Logic	Key Pathways/Molecules	Limitations/Conclusion	Supporting References
Major Ginsenosides (Rb1, Rb2, Rg1, Rd)	3T3-L1 preadipocytes; DIO C57BL/6J mice	Direct Activation	Signal Transduction	AMPK, PKA, β3-AR, PGC-1α	Well-validated; effects may vary based on metabolic background.	[31,32,33,34,35,36,37,38,39,40,41,42]
Rare Ginsenosides (F1, Rg3)	ob/ob mice; DIO C57BL/6J mice	Direct & Indirect	Signaling/Metabolic Reprogramming	cAMP/PKA, AMPK/mTORC1	Primarily WAT-specific; requires receptor-binding assays to confirm causality.	[44,45,46,47,48,49]
Polysaccharides/Dietary Fiber	DIO C57BL/6J mice	Indirect Regulation	Environment Optimization	Gut microbiota-metabolite axis	Mostly correlative; lacks direct evidence for canonical thermogenic marker induction.	[50,51,52,53,54]
Microbial Metabolites (e.g., MA)	Antibiotic-treated mice; FMT-recipient C57BL/6J mice	Indirect Sensitization	Signal Enhancement	β-AR sensitization	Highlights systemic synergy; currently associative, lacking direct receptor-interaction proof.	[69,70,71,82,90]
Ginsenoside Rh4 (Gut-Liver Axis)	DIO C57BL/6J mice; db/db diabetic mice	Indirect Regulation	Organ Cross-talk	Bile acids, FXR, PPARα	Inter-organ communication; no direct evidence of action on adipocytes.	[63,75,95,96,97,98]

Note: The classification of evidence is based on currently available preclinical studies. “Direct activation” refers to effects supported by experimental data demonstrating engagement of established intracellular signaling pathways in adipocytes. “Indirect sensitization” describes regulatory influences that appear to operate through modulation of the broader metabolic environment, often involving gut–organ axis-mediated interactions rather than direct adipocyte receptor activation.

### 4.3. Ginseng Participates in Energy Metabolism Related to Adipose Tissue Browning Through the Gut–Brain Axis

The gut–brain axis (GBA) represents a bidirectional communication network linking the gastrointestinal tract and the central nervous system through neural, endocrine, and immune pathways. Its core components include the enteric nervous system, the autonomic nervous system, the hypothalamic–pituitary–adrenal (HPA) axis, and the gut microbiota, which together coordinate the regulation of energy homeostasis, feeding behavior, and metabolic adaptation [99,100]. Within this axis, gut microbiota-derived metabolites serve as key signaling intermediates that convey peripheral nutritional information to central energy-sensing circuits. Among these metabolites, bile acids can activate the TGR5 receptor and suppress the activity of orexigenic agouti-related peptide/neuropeptide Y (AgRP/NPY) neurons in the hypothalamus, thereby influencing food intake behavior [101]. In parallel, SCFAs produced in the gut bind to free fatty acid receptors FFAR2 and FFAR3, stimulating the secretion of gut-derived hormones such as GLP-1, PYY, and leptin. These hormonal signals are transmitted to the arcuate nucleus (ARC) of the hypothalamus, partly via vagal pathways, and are associated with inhibition of NPY/AgRP neurons and activation of proopiomelanocortin/cocaine- and amphetamine-regulated transcript (POMC/CART) neurons. Through these central mechanisms, SCFAs contribute to the regulation of appetite, energy intake, and the balance between energy consumption and expenditure, thereby providing neuroendocrine support for adipose tissue browning-related energy metabolism [102,103,104,105].

In the context of ginseng research, accumulating evidence suggests that ginseng may influence energy metabolism and central feeding control through modulation of the gut–brain axis. Previous studies have shown that ginseng and its bioactive components can downregulate hypothalamic NPY expression and modulate circulating levels of metabolic hormones, including leptin and insulin, effects that are associated with reduced energy intake and decreased fat accumulation [106]. In addition, ginseng has been reported to attenuate hypothalamic inflammatory responses and regulate neurotransmitter metabolism, including glutamate, glutamine, glycine, and γ-aminobutyric acid (GABA), thereby supporting neuronal integrity and improving central energy-sensing homeostasis [107]. Further evidence indicates that black ginseng extract can markedly reduce adipose tissue accumulation and improve hypothalamic function in high-fat diet-fed mouse models. Moreover, ginsenoside Rb1 has been shown to suppress the expression of hypothalamic inflammatory markers, such as IL-6, IL-1β, and phosphorylated inhibitor of κB kinase (p-IKK), while restoring insulin signaling pathway activity under obese conditions [108]. Through these system-level effects, ginseng may alleviate obesity-related metabolic abnormalities by modulating hypothalamic inflammation and central metabolic signaling.

**Figure 6:**
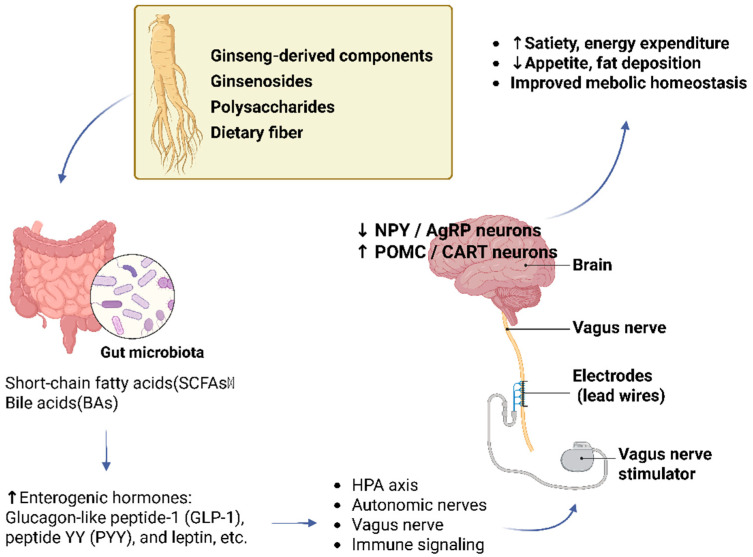
Potential mechanisms by which ginseng promotes adipose tissue browning through the gut–brain axis (BioRender.com. Agreement number: JG29BE3Y3U). The circular arrows illustrate the communication loop where gut-derived metabolites (SCFAs and bile acids) act as signaling messengers. Arrows pointing to the brain represent the modulation of central appetite-regulating neurons (NPY/AgRP inhibition and POMC/CART activation) via neural and endocrine pathways to support systemic thermogenic output.

Taken together, the available evidence suggests that ginseng may remodel energy-sensing and neuroendocrine regulatory states along the gut–brain axis by coordinating gut-derived metabolic signals with central appetite-regulating networks. As illustrated in Figure 6, ginseng may indirectly promote adipose tissue browning through modulation of gut-derived metabolites and central neuroendocrine regulation. Such central metabolic control not only contributes to reduced energy intake but may also support enhanced thermogenic function of adipose tissue at the systemic level by influencing sympathetic nervous system output and improving peripheral tissue responsiveness to energy-related signals. Through these integrated mechanisms, ginseng may indirectly participate in adipose tissue browning-associated metabolic processes and exert potential beneficial effects on obesity and obesity-related metabolic disturbances.

## 5. Role of Fermentation in Enhancing Ginseng-Induced White Adipose Tissue Browning

Recent studies indicate that processing methods can influence not only the content and composition of ginseng bioactive constituents but also the pathways and hierarchical levels through which ginseng participates in the regulation of energy metabolism. Among these approaches, fermentation—long regarded as one of the most effective strategies for enhancing the functionality of medicinal and edible foods—has attracted particular attention. Fermented ginseng may influence adipose tissue browning through both direct modulation of intracellular signaling and indirect microbiota-mediated mechanisms. Compared with unprocessed ginseng, fermented preparations have shown differences in regulatory patterns in experimental models of WAT browning. These differences may relate to alterations in ginsenoside composition, metabolite profiles, and bioavailability introduced during fermentation, rather than simply reflecting stronger activity. By systematically altering the structural forms of ginsenosides and their in vivo metabolic behavior, fermentation appears to shift ginseng from a predominantly local, target-oriented mode of action toward a broader, multi-level involvement in energy metabolic regulation [109]. Several preclinical studies have shown that fermented ginseng is linked to sustained AMPK activation and continued expression of thermogenic genes [110]. In some models, these effects seem to last longer and involve a wider range of metabolic markers than those seen with unprocessed ginseng. Together, these findings suggest that processing—especially fermentation—may influence the metabolic effects of ginseng. However, the reasons for these differences are still unclear. It remains uncertain whether fermentation enhances the inherent activity of certain components, changes their bioavailability or metabolic transformation in vivo, or affects adaptive stress and signaling responses. Clarifying these issues will require well-controlled, direct comparative studies.

### 5.1. Direct Adipocyte-Level Mechanisms

At the level of adipose tissue, existing studies consistently report that fermented ginseng supports thermogenic signaling pathways, including β_3_-AR-related and AMPK-associated mechanisms, which are linked to browning-related transcriptional activity in experimental models [111]. In parallel, modulation of the AMPK signaling pathway by fermented ginseng contributes to the maintenance of key transcriptional regulators, including PGC-1α and PRDM16, providing a molecular basis for mitochondrial biogenesis and fatty acid oxidation [112]. Compared with native ginseng, the effects of fermented ginseng on these canonical browning pathways tend to manifest as more sustained and stable signaling support rather than transient activation, a characteristic that appears to be closely associated with structural transformation of its bioactive components and altered in vivo exposure profiles.

### 5.2. Indirect Microbiota-Dependent Mechanisms

Beyond its direct effects on adipose tissue, regulation at the level of the gut microbiota represents another distinguishing feature of fermented ginseng compared with its non-fermented counterpart. Fermented ginseng has been reported to influence gut microbial composition, including increased abundance of certain genera such as *Akkermansia* and *Lactobacillus*. Although native ginseng can also affect microbiota structure, fermentation may modify the spectrum of bioactive compounds available to the gut, thereby shaping microbial responses in a distinct manner [113,114]. Alterations in gut microbial composition further influence the production of SCFAs and functional bile acids, which act as microbial-derived metabolites capable of modulating energy metabolism and thermogenesis through signaling pathways such as GPR43 and TGR5 in both the liver and adipose tissue [115,116]. Accordingly, fermented ginseng appears to support WAT browning not solely through direct stimulation of adipocytes but also by establishing a sustained regulatory axis linking the gut microbiota, microbial metabolites, and adipose tissue.

In addition, fermented ginseng may indirectly facilitate the browning process by improving the local microenvironment of adipose tissue. Available evidence indicates that fermented ginseng intervention reduces the expression of pro-inflammatory factors in adipose tissue, suppresses the activity of lipogenesis-related transcription factors, and improves insulin sensitivity [117,118,119]. Optimization of this microenvironment is thought to alleviate constraints imposed by metabolic stress on WAT browning, thereby enhancing the overall responsiveness of adipose tissue to browning stimuli. This process is also considered to contribute to the maintenance and stability of the browning phenotype [120].

Taken together, current evidence suggests that fermented ginseng may influence WAT browning through effects at multiple levels, including modulation of adipocyte signaling and microbiota-associated systemic regulation [109,110,111,112,121,122]. However, the relative contribution of these direct and indirect pathways under physiologically relevant conditions remains unclear. From a translational perspective, most supporting data derive from preclinical models. Although fermented ginseng has shown metabolic effects in experimental systems, direct clinical comparisons with native preparations are limited. In addition, factors such as dose standardization, long-term safety, bioavailability, and inter-individual variability in gut microbiota composition may substantially influence clinical outcomes. These considerations highlight the need for carefully designed human studies to determine the practical feasibility of fermented ginseng in metabolic health management.

## 6. Limitations and Perspectives

While the preceding sections have examined mechanistic interpretations of ginseng-associated metabolic regulation, several practical and translational limitations remain. A major constraint arises from the experimental systems currently used to evaluate metabolic outcomes. Much of the available evidence derives from in vitro adipocyte models or diet-induced obese rodent studies [30,55]. Although these systems help identify intracellular targets and pathway responsiveness, they do not capture long-term metabolic adaptation under habitual dietary exposure. Compound concentrations employed in preclinical models frequently exceed nutritionally achievable levels, and intervention durations are short relative to human metabolic timescales [26,28,109]. Direct extrapolation to dietary contexts is therefore uncertain.

Limitations in functional validation further complicate interpretation. Many studies rely primarily on transcriptional or protein-level markers to infer metabolic outcomes [31,33,42], yet changes in gene expression do not necessarily correspond to measurable alterations in substrate oxidation or energy expenditure. Without direct assessment of mitochondrial respiration, whole-body metabolic rate, or lipid flux, the physiological relevance of observed molecular responses remains unclear. Integrating functional metabolic measurements with molecular analyses would substantially strengthen causal interpretation.

These issues become more apparent when considering translation to human physiology. Clinical investigations of ginseng have largely focused on glycemic control, lipid metabolism, or inflammatory markers [3,4,21], whereas direct evaluation of adipose thermogenic remodeling is rare. Imaging technologies such as PET-CT and MRI allow non-invasive assessment of adipose metabolic activity [6,11,22], but imaging-based findings require tissue-level molecular validation to confirm sustained remodeling [7,10]. Combining metabolic imaging with adipose biopsy would provide more definitive evidence of thermogenic adaptation. Translational relevance also depends on accurate characterization of compound exposure. Pharmacokinetic studies are necessary to define absorption, metabolic transformation, and tissue distribution of ginsenosides and fermentation-derived metabolites under realistic intake conditions [26,28,109,121]. Stable isotope tracer methodologies can complement these efforts by quantifying substrate oxidation and lipid turnover in vivo, thereby linking signaling changes to metabolic flux [15,18].

Consideration of inter-individual variability is equally important. Differences in gut microbiota composition and baseline metabolic phenotype may influence responsiveness to ginseng intervention. Clinical trials incorporating microbiome profiling and circulating metabolite analysis could help identify responder subgroups and clarify whether metabolic effects are context-dependent. Advancing this field will therefore depend on physiologically grounded experimental paradigms, rigorous functional validation, and carefully designed translational studies. Progress will be determined less by additional pathway identification and more by establishing whether molecular observations translate into sustained metabolic outcomes under realistic human conditions.

## 7. Conclusions

Findings from cellular and animal studies compiled in this review indicate that ginseng-derived components are capable of influencing energy-sensing pathways, mitochondrial function, and systemic metabolic signaling, thereby affecting the adipose tissue’s response to energy demands. Consequently, ginseng-induced browning should be regarded as a form of metabolic regulation rather than a direct activation of thermogenic programs. The increases in thermogenic markers reported in experimental models may, in fact, reflect alterations in metabolic regulation and cellular adaptability. These observations underscore the importance of considering adipose tissue responses within the broader context of systemic metabolic regulation. Interactions involving the gut microbiota, bile acid signaling, and crosstalk among metabolic organs are all likely contributing factors to the observed effects. From this perspective, ginseng serves as a useful model for understanding how dietary bioactive compounds can influence adipose tissue metabolism through coordinated physiological regulation. From a translational medicine standpoint, these findings suggest potential relevance for metabolic health and obesity-related disorders. However, it must be emphasized that the majority of currently available evidence has been derived from preclinical cellular or animal models. Further comprehensive investigations are still required to elucidate causal mechanisms, determine physiological relevance under actual dietary intake conditions, and assess whether these regulatory effects can translate into sustained metabolic benefits for humans. Therefore, substantial work remains before these findings can be applied to clinical or nutritional practice.

In summary, this review has not only systematically consolidated research progress on ginseng in promoting white adipose browning, but more importantly, it has provided a “model” for understanding how dietary components can influence metabolism through systemic physiological regulation, thereby indicating feasible directions for future fundamental research and translational applications of functional foods.

## Figures and Tables

**Table 1:** Effects of ginseng on gut microbiota composition of mice and their potential metabolic functions.

Genus	Metabolic Function	Model	Experimental Animals	Ginseng Component
*Akkermansia* ↑	Gut barrier integrity, insulin sensitivity, anti-inflammatory effects	DIO	C57BL/6J	Red ginseng extract, red ginseng dietary fiber, ginsenoside Rb1
*Roseburia* ↑	Butyrate production, energy metabolism	DIO	C57BL/6J	Red ginseng extract
*Monoglobus* ↑	Dietary fiber degradation	DIO	C57BL/6J	Red ginseng dietary fiber
*Parasutterella* ↑	Regulation of bile acid and lipid metabolism	DIO	C57BL/6J	Red ginseng extract, ginsenoside Rb1
*Bacteroides* ↑	Polysaccharide utilization, SCFA production	DIO	C57BL/6J	Ginsenoside Rb1, ginseng polysaccharides
*Parabacteroides* ↑	Anti-inflammatory effects, lipid metabolism improvement	DIO/IBS	C57BL/6J	Red ginseng
*Faecalibacterium* ↑	Butyrate production, anti-inflammatory effects	DIO	C57BL/6J	Ginseng polysaccharides (in some studies)
*Anaerostipes* ↑	Butyrate production	DIO	C57BL/6J	Ginseng polysaccharides
*Blautia* ↑	Energy homeostasis, SCFA production	DIO	C57BL/6J	Red ginseng extract
*Butyricicoccus* ↑	Gut barrier function, butyrate production	DIO	C57BL/6J	Ginseng polysaccharides
*Lactobacillus* ↑	Metabolic and inflammatory regulation	DIO	db/db mice (Lepr^−^/^−^, C57BLKS/J)	Red ginseng, ginseng polysaccharides
*Bifidobacterium* ↑	Anti-inflammatory effects, metabolic improvement	DIO	C57BL/6J	Ginseng polysaccharides
*Allobaculum* ↑	Associated with lean phenotype	DIO	C57BL/6J	Red ginseng extract
*Desulfovibrio* ↓	LPS and H_2_S production, pro-inflammatory	DIO	C57BL/6J	Red ginseng extract, red ginseng dietary fiber
*Bilophila* ↓	Bile acid-dependent pro-inflammatory bacterium	DIO	C57BL/6J	Red ginseng dietary fiber
*Oscillibacter* ↓	Associated with insulin resistance	DIO	C57BL/6J	Red ginseng extract
*Ruminococcus* ↓	Mucin degradation, inflammation	DIO	C57BL/6J	Red ginseng extract
*Alistipes* ↓	Obesity- and inflammation-associated	DIO	C57BL/6J	Red ginseng dietary fiber
*Enterobacter* ↓	Source of endotoxin	DIO	C57BL/6J	Ginseng polysaccharides
*Escherichia* ↓	LPS production, metabolic endotoxemia	DIO	C57BL/6J	Ginseng polysaccharides
*Klebsiella* ↓	Inflammation, fatty liver-associated	DIO	C57BL/6J	Red ginseng
*Helicobacter* ↓	Pro-inflammatory	DIO	C57BL/6J	Red ginseng
*Prevotella* ↓	Positively associated with insulin resistance	DIO	C57BL/6J	Red ginseng

Note: ↑ indicates increased relative abundance of the indicated gut microbial genus, whereas ↓ indicates decreased relative abundance.

## Data Availability

No new data were created or analyzed in this study. Data sharing is not applicable to this article.

## References

[1] World Obesity Federation (2024). World Obesity Atlas 2024.

[2] Blüher M. (2019). Obesity: Global epidemiology and pathogenesis. Nat. Rev. Endocrinol..

[3] Afshin A., Forouzanfar M.H., Reitsma M.B. (2017). Health effects of overweight and obesity in 195 countries over 25 years. N. Engl. J. Med..

[4] Saltiel A.R., Olefsky J.M. (2017). Inflammatory mechanisms linking obesity and metabolic disease. J. Clin. Investig..

[5] Rosen E.D., Spiegelman B.M. (2014). What we talk about when we talk about fat. Cell.

[6] Cannon B., Nedergaard J. (2004). Brown adipose tissue: Function and physiological significance. Physiol. Rev..

[7] Kajimura S., Spiegelman B.M., Seale P. (2015). Brown and beige fat: Physiological roles beyond heat generation. Cell Metab..

[8] Wu J., Boström P., Sparks L.M. (2012). Beige adipocytes are a distinct type of thermogenic fat cell in mouse and human. Cell.

[9] Seale P., Bjork B., Yang W. (2008). PRDM16 controls a brown fat/skeletal muscle switch. Nature.

[10] Harms M., Seale P. (2013). Brown and beige fat: Development, function and therapeutic potential. Nat. Med..

[11] Cypess A.M., Weiner L.S., Roberts-Toler C. (2015). Activation of human brown adipose tissue by a β3-adrenergic receptor agonist. Cell Metab..

[12] Bartness T.J., Shrestha Y.B., Vaughan C.H. (2014). Neural innervation of white adipose tissue and the control of lipolysis. Am. J. Physiol. Regul. Integr. Comp. Physiol..

[13] Xu B., Goulding E.H., Zang K. (2003). Brain-derived neurotrophic factor regulates energy balance downstream of melanocortin-4 receptor. Nat. Neurosci..

[14] Enerbäck S., Jacobsson A., Simpson E.M. (1997). Mice lacking mitochondrial uncoupling protein are cold-sensitive but not obese. Nature.

[15] Bianco A.C., Salvatore D., Gereben B. (2002). Deiodinases: Implications of the local control of thyroid hormone action. J. Clin. Investig..

[16] Puigserver P., Spiegelman B.M. (2003). Peroxisome proliferator-activated receptor-γ coactivator 1α (PGC-1α). Endocr. Rev..

[17] Kajimura S., Seale P., Tomaru T. (2009). Initiation of myoblast to brown fat switch by a PRDM16–C/EBP-β transcriptional complex. Nature.

[18] Hardie D.G. (2008). AMPK: A key regulator of energy balance in the single cell and the whole organism. Int. J. Obes..

[19] Hardie D.G., Ross F.A., Hawley S.A. (2012). AMPK: A nutrient and energy sensor that maintains energy homeostasis. Nat. Rev. Mol. Cell Biol..

[20] Herzig S., Shaw R.J. (2018). AMPK: Guardian of metabolism and mitochondrial homeostasis. Nat. Rev. Mol. Cell Biol..

[21] Arch J.R.S. (2002). The discovery of drugs for obesity, the metabolic effects of leptin and β3-adrenoceptor agonists. Int. J. Obes..

[22] Carey A.L., Formosa M.F., Van Every B. (2013). Ephedrine activates brown adipose tissue in lean but not obese humans. Diabetes.

[23] Attele A.S., Wu J.A., Yuan C.S. (1999). Ginseng pharmacology: Multiple constituents and multiple actions. Biochem. Pharmacol..

[24] Luo H.-Y., Fang J., Zhang W.-H., Chan K.-C., Chan Y.-M., Dong C.-X., Li S.-L., Lyu A.-P., Xu J. (2025). Dissecting the anti-obesity components of ginseng: How ginseng polysaccharides and ginsenosides target gut microbiota to suppress high-fat diet-induced obesity. J. Adv. Res..

[25] Kim J.H. (2012). Cardiovascular diseases and Panax ginseng: A review. J. Ginseng Res..

[26] Christensen L.P. (2009). Ginsenosides: Chemistry, biosynthesis, analysis, and potential health effects. Adv. Food Nutr. Res..

[27] Hou M., Wang R., Zhao S. (2021). Ginsenosides in Panax genus and their biosynthesis. Acta Pharm. Sin. B.

[28] Kim T.H. (2024). Ginsenosides for the Treatment of Insulin Resistance and Diabetes: Therapeutic Perspectives and Mechanistic Insights. J. Ginseng Res..

[29] Fan W., Fan L., Wang Z., Mei Y., Liu L., Li L., Yang L., Wang Z. (2024). Rare ginsenosides: A unique perspective of ginseng research. J. Adv. Res..

[30] Thyagarajan B., Foster M.T. (2017). Beiging of White Adipose Tissue as a Therapeutic Strategy for Weight Loss in Humans. Horm. Mol. Biol. Clin. Investig..

[31] Shen L., Xiong Y., Wang D.Q. (2013). Ginsenoside Rb1 reduces obesity by activating AMPK signaling pathway. J. Ethnopharmacol..

[32] Xu M.Y., Lee S.Y., Kang S.S., Kim Y.S. (2009). Antioxidant and anti-adipogenic activities of ginsenoside Rb1. Phytother. Res..

[33] Zhou Y., Wang S., Ying H. (2018). Ginsenoside Rb1 induces beige adipocyte formation via AMPK signaling. Biochem. Biophys. Res. Commun..

[34] Shen T., Lee J.H., Park M.H. (2012). Ginsenoside Rb1 suppresses hepatic gluconeogenesis and inflammation. Biochem. Pharmacol..

[35] Wang Y., Liu J., Ma A., Chen Y. (2015). Ginsenoside Rb1 attenuates inflammation by inhibiting NF-κB/NLRP3 signaling. Int. Immunopharmacol..

[36] Liu Y., Wei J., Gao Y. (2020). Ginsenoside Rb2 regulates lipid metabolism through AMPK–ULK1–mTOR pathway. Phytomedicine.

[37] Machado S.A., Pasquarelli-do-Nascimento G., da Silva D.S. (2022). Browning of the White Adipose Tissue Regulation: New Insights into Nutritional and Metabolic Relevance in Health and Diseases. Nutr. Metab..

[38] Kim J.H., Hahm D.H., Yang D.C. (2014). Ginsenoside Rb2 activates PI3K/Akt signaling and improves energy metabolism. J. Ginseng Res..

[39] Lee Y.S., Kim W.S., Kim K.H. (2012). Ginsenoside Rg1 activates AMPK and enhances fatty acid oxidation. Metabolism.

[40] Lee K., Seo Y.-J., Song J.-H. (2018). Ginsenoside Rg1 Promotes Browning by Inducing UCP1 Expression and Mitochondrial Activity in 3T3-L1 and Subcutaneous White Adipocytes. J. Ginseng Res..

[41] Hong Y., Lin Y., Si Q., Yang L. (2016). Ginsenoside Rd promotes thermogenic gene expression via cAMP/PKA signaling. Phytother. Res..

[42] Yao L., Han Z., Zhao G., Yanfang X., Zhou X., Dai R., Han M., Wang Z., Xin R., Wang S. (2020). Ginsenoside Rd Ameliorates High Fat Diet-Induced Obesity by Enhancing Adaptive Thermogenesis in a cAMP-Dependent Manner. Obesity.

[43] Nag S.A., Qin J.J., Wang W. (2012). Ginsenosides as anticancer agents: Structure–activity relationship. Front. Pharmacol..

[44] Meng Y., Li W., Hu C. (2023). Ginsenoside F1 Administration Promotes UCP1-Dependent Fat Browning and Ameliorates Obesity-Associated Insulin Resistance. Food Sci. Hum. Wellness.

[45] Shin J., Lee Y., Ju S.H., Jung Y.J., Sim D., Lee S.-J. (2024). Unveiling the Potential of Natural Compounds: A Comprehensive Review on Adipose Thermogenesis Modulation. Int. J. Mol. Sci..

[46] Wu Y., Chen Y., Xu J. (2019). Ginsenoside Rg1 promotes browning of white adipocytes. Biochem. Biophys. Res. Commun..

[47] Hwang J.T., Lee M.S., Kim H.J. (2009). Anti-obesity effect of ginsenoside Rg3 via AMPK activation. Biochem. Pharmacol..

[48] Liu W., Zhang S.-X., Ai B., Pan H., Zhang D., Jiang Y., Hu L., Sun L., Chen Z.-S., Lin L. (2021). Ginsenoside Rg3 Promotes Cell Growth through Activation of mTORC1. Front. Cell Dev. Biol..

[49] Lee J.H., Kim M.H., Kim J.H. (2020). Ginsenoside Rg3 suppresses adipogenesis and promotes energy expenditure. Int. J. Mol. Sci..

[50] Sun Y., Li T., Xie J. (2018). Structural characterization and bioactivity of ginseng polysaccharides. Carbohydr. Polym..

[51] Wang J., Zhou Y., Yu Y., Wang Y., Xue D., Zhou Y., Li X. (2023). A Ginseng-Derived Rhamnogalacturonan I (RG-I) Pectin Promotes Longevity via TOR Signalling in Caenorhabditis elegans. Carbohydr. Polym..

[52] Li S., Chen C., Wang J. (2019). Steamed ginseng polysaccharide GPS-1 activates AMPK pathway. J. Funct. Foods.

[53] Slavin J. (2005). Dietary fiber and body weight. Nutrition.

[54] Canfora E.E., Jocken J.W., Blaak E.E. (2015). Short-chain fatty acids in control of body weight and insulin sensitivity. Nat. Rev. Endocrinol..

[55] Moreno-Navarrete J.M., Fernández-Real J.M. (2019). The gut microbiota modulates both browning of white adipose tissue and the activity of brown adipose tissue. Rev. Endocr. Metab. Disord..

[56] Qi L.W., Wang C.Z., Yuan C.S. (2011). Isolation and analysis of ginseng: Advances and challenges. Nat. Prod. Rep..

[57] Peng D., Wang H., Qu C., Xie L.H., Wicks S.M., Xie J.T. (2012). Ginsenoside Re: Its chemistry, metabolism and pharmacokinetics. Chin. Med..

[58] Calabrese E.J. (2020). Hormesis and ginseng: Ginseng mixtures and individual constituents commonly display hormesis dose responses, especially for neuroprotective effects. Molecules.

[59] Li Z., Wang Y., Xu Q., Ma J., Li X., Tian Y., Wen Y., Chen T. (2023). Ginseng and Health Outcomes: An Umbrella Review. Front. Pharmacol..

[60] Zhang L. (2020). Ginseng oligopeptides attenuate obesity-related inflammation. Phytomedicine.

[61] Lagouge M., Argmann C., Gerhart-Hines Z. (2006). Resveratrol improves mitochondrial function and protects against metabolic disease by activating SIRT1 and PGC-1α. Cell.

[62] Ummarino D. (2017). Matrix turnover linked to dietary weight loss. Nat. Rev. Rheumatol..

[63] den Besten G. (2013). The role of short-chain fatty acids in the interplay between diet, gut microbiota, and host energy metabolism. J. Lipid Res..

[64] Canfora E.E., Meex R.C.R., Venema K., Blaak E.E. (2019). Gut microbial metabolites in obesity, NAFLD and T2DM. Nat. Rev. Endocrinol..

[65] Kimura I., Inoue D., Hirano K., Tsujimoto G. (2014). The SCFA receptor GPR43 and energy metabolism. Front. Endocrinol..

[66] Byrne C., Chambers E.S., Morrison D.J., Frost G. (2015). The role of short-chain fatty acids in appetite regulation and energy homeostasis. Int. J. Obes..

[67] May K.S., den Hartigh L.J. (2021). Modulation of Adipocyte Metabolism by Microbial Short-Chain Fatty Acids. Nutrients.

[68] Li Z., Yi C.X., Katiraei S., Kooijman S., Zhou E., Chung C.K., Gao Y., van den Heuvel J.K., Meijer O.C., Berbée J.F.P. (2018). MCT1-dependent transport of propionate promotes white adipose tissue browning. Mol. Metab..

[69] Tolhurst G., Heffron H., Lam Y.S., Parker H.E., Habib A.M., Diakogiannaki E., Cameron J., Grosse J., Reimann F., Gribble F.M. (2012). Short-chain fatty acids stimulate glucagon-like peptide-1 secretion via FFAR2. Diabetes.

[70] Gao Z., Yin J., Zhang J., Ward R.E., Martin R.J., Lefevre M., Cefalu W.T., Ye J. (2009). Butyrate improves insulin sensitivity and increases energy expenditure in mice. Diabetes.

[71] Davie J.R. (2003). Inhibition of histone deacetylase activity by butyrate. J. Nutr..

[72] Li X., Wang Y., Wang K., Wu Y., Liu J., Chen S., Zhou Y. (2020). Ginsenosides regulate bile acid metabolism via FXR/CYP7A1 signaling to improve metabolic disorders. Phytomedicine.

[73] St-Onge M.P., Jones P.J.H. (2002). Physiological effects of medium-chain triglycerides: Potential agents in the prevention of obesity. J. Nutr..

[74] Nagao K., Yanagita T. (2009). Medium-Chain Fatty Acids: Functional Lipids for the Prevention and Treatment of the Metabolic Syndrome. Pharmacol. Res..

[75] Zhang J., Lv W., Liu X., Sun Z., Zeng M., Kang J., Zhang Q., Liu F., Ma S., Su J. (2024). Ginsenoside Rh4 prevents endothelial dysfunction as a novel AMPK activator. Br. J. Pharmacol..

[76] Chen Z., Zhang Z., Liu J. (2022). Gut microbiota: Therapeutic targets of ginseng against multiple disorders and ginsenoside transformation. Front. Cell. Infect. Microbiol..

[77] Bai X., Duan Z., Deng J. (2025). Ginsenoside Rh4 inhibits colorectal cancer via the modulation of gut microbiota-mediated bile acid metabolism. J. Adv. Res..

[78] Wang J., Li Y., Zhao X. (2021). Ginsenoside Rg1 alleviates obesity by modulating bile acid profiles and gut microbiota. J. Ginseng Res..

[79] Ding L., Yang L., Wang Z., Huang W. (2015). Bile Acid Nuclear Receptor FXR and Digestive System Diseases. Acta Pharm. Sin. B.

[80] Watanabe M., Houten S.M., Mataki C., Christoffolete M.A., Kim B.W., Sato H., Messaddeq N., Harney J.W., Ezaki O., Kodama T. (2006). Bile acids induce energy expenditure by promoting intracellular thyroid hormone activation. Nature.

[81] Zhang Y., Lee F.Y., Barrera G. (2006). Activation of FXR improves lipid metabolism and insulin sensitivity. Cell Metab..

[82] Wang J., Li Y., Cheung K., Zheng X. (2023). Bile Acid Signaling in the Regulation of Whole Body Metabolic and Immunological Homeostasis. Sci. China Life Sci..

[83] Li X., Yao Y., Yu C. (2022). Modulation of PPARα-thermogenesis gut microbiota interactions in obese mice administrated with zingerone. J. Sci. Food Agric..

[84] den Besten G., Bleeker A., Gerding A. (2015). Short-chain fatty acids protect against diet-induced obesity via a PPARγ-dependent switch. J. Lipid Res..

[85] Wang Y., Tang Y., He Z.H. (2021). Slit3 secreted from M2-like macrophages increases sympathetic activity and thermogenesis in adipose tissue. Nat. Metab..

[86] Nguyen K.D., Qiu Y., Cui X. (2011). Alternatively activated macrophages produce catecholamines to sustain adaptive thermogenesis. Nature.

[87] Aryal B., Singh A.K., Zhang X., Varela L., Rotllán N., Goedeke L., Chaube B., Camporez J.P., Vatner D.F., Horváth T.L. (2018). Absence of ANGPTL4 in Adipose Tissue Improves Glucose Tolerance and Attenuates Atherogenesis. JCI Insight.

[88] Kleiner S., Spiegelman B.M. (2013). PPARγ coactivator 1α: Transcriptional control of mitochondrial biogenesis. Endocr. Rev..

[89] Garcia D., Shaw R.J. (2017). AMPK: Mechanisms of cellular energy sensing and restoration of metabolic balance. Molec. Cell.

[90] Quan L.H., Zhang C., Dong M. (2019). Myristoleic acid produced by enterococci reduces obesity through brown fat activation. Nat. Commun..

[91] Bäckhed F., Ding H., Wang T., Hooper L.V., Koh G.Y., Nagy A., Semenkovich C.F., Gordon J.I. (2004). The Gut Microbiota as an Environmental Factor That Regulates Fat Storage. Proc. Natl. Acad. Sci. USA.

[92] Iván J., Major E., Sipos A. (2017). The short-chain fatty acid propionate inhibits adipogenic differentiation of human chorion-derived mesenchymal stem cells through free fatty acid receptor 2. Stem Cells Dev..

[93] Zhu X., Wang X., Wang J. (2024). Intermittent fasting-induced Orm2 promotes adipose browning via the GP130/IL23R-p38 cascade. Adv. Sci..

[94] Kim K.H., Kim Y.H., Son J.E. (2017). Intermittent fasting promotes adipose thermogenesis and metabolic homeostasis via VEGF-mediated alternative activation of macrophage. Cell Res..

[95] Alhamoud Y., Abudumijiti T., Wu J. (2024). Stimulation of non-shivering thermogenesis by bioactive compounds: A focus on gut microbiota-mediated mechanisms. Trends Food Sci. Technol..

[96] Zhao L., Sui M., Zhang T. (2023). The interaction between ginseng and gut microbiota. Front. Nutr..

[97] Zhang Z., He Y., Zhao M. (2024). Qinlian Hongqu decoction modulates FXR/TGR5/GLP-1 pathway to improve insulin resistance in NAFLD mice: Bioinformatics and experimental study. ACS Omega.

[98] Chiang J.Y.L. (2013). Bile acid metabolism and signaling. Compr. Physiol..

[99] Yang S., Duan Z., Zhang S. (2023). Ginsenoside Rh4 improves hepatic lipid metabolism and inflammation in a model of NAFLD by targeting the gut–liver axis and modulating FXR signaling pathway. Foods.

[100] Turnbaugh P.J., Ley R.E., Mahowald M.A. (2006). An obesity-associated gut microbiome with increased capacity for energy harvest. Nature.

[101] Lynch S.V., Pedersen O. (2016). The human intestinal microbiome in health and disease. N. Engl. J. Med..

[102] Brestoff J.R., Artis D. (2015). Immune regulation of metabolic homeostasis in health and disease. Cell.

[103] Silva A.I., Direito M., Pinto-Ribeiro F. (2023). Effects of intermittent fasting on regulation of metabolic homeostasis: A systematic review and meta-analysis in health and metabolic-related disorders. J. Clin. Med..

[104] Dijk W., Kersten S. (2014). Regulation of lipid metabolism by angiopoietin-like proteins. Curr. Opin. Lipidol..

[105] Lin J., Handschin C., Spiegelman B.M. (2005). Metabolic control through the PGC-1 family. Cell Metab..

[106] Cryan J.F., Dinan T.G. (2012). Mind-altering microorganisms: The impact of the gut microbiota on brain and behaviour. Nat. Rev. Neurosci..

[107] Frost G., Sleeth M.L., Sahuri-Arisoylu M. (2014). The short-chain fatty acid acetate reduces appetite via a central homeostatic mechanism. Nat. Commun..

[108] Perry R.J., Peng L., Barry N.A. (2016). Acetate mediates a microbiome–brain–β-cell axis to promote metabolic syndrome. Nature.

[109] Wang W., Xue Y.J., Zhou J. (2024). Exploring the antimicrobial activity of rare ginsenosides and the progress of their related pharmacological effects. Phytomedicine.

[110] Kim D.H. (2017). Gut microbiota-mediated pharmacokinetics of ginseng saponins. J. Ginseng Res..

[111] Feng F., Ko H.A., Truong T.M.T., Song W.J., Ko E.J., Kang I. (2024). Ginsenoside Rg3, enriched in red ginseng extract, improves lipopolysaccharide-induced suppression of brown and beige adipose thermogenesis with mitochondrial activation. Sci. Rep..

[112] Nan B., Liu Y., You Y. (2018). Protective effects of enhanced minor ginsenosides in Lactobacillus fermentum KP-3–fermented ginseng in mice fed a high-fat diet. Food Funct..

[113] Kang D., Li Z., Ji G.E. (2018). Anti-obesity effects of a mixture of fermented ginseng, Bifidobacterium longum BORI, and Lactobacillus paracasei CH88 in high-fat diet-fed mice. J. Microbiol. Biotechnol..

[114] Bai J., Zhu Z., Luo W. (2025). Microbial fermentation affects the structure–activity relationship of bioactive compounds in ginseng and its applications in fermentation products: A review. Foods.

[115] Chambers E.S., Viardot A., Psichas A., Morrison D.J., Murphy K.G., Zac-Varghese S.E., MacDougall K., Preston T., Tedford C., Finlayson G.S. (2015). Effects of targeted delivery of propionate to the human colon on appetite regulation, body weight maintenance, and adiposity in overweight adults. Gut.

[116] Shin Y.-J., Lee D.-Y., Kim J.Y. (2023). Effect of fermented red ginseng on gut microbiota dysbiosis- or immobilization stress-induced anxiety, depression, and colitis in mice. J. Ginseng Res..

[117] Oh J.S., Lee S.R., Hwang K.T. (2014). Anti-obesity effects of the dietary combination of fermented red ginseng with levan in a high-fat diet mouse model. Phytother. Res..

[118] Kho M.C., Lee Y.J., Park J.H. (2016). Fermented red ginseng potentiates improvement of metabolic dysfunction in metabolic syndrome rat models. Nutrients.

[119] Yang C., Zhang Y., Jia M. (2025). Fermentation interactions and lipid-lowering potential of Monascus purpureus-fermented ginseng. Food Biosci..

[120] Zhou Q., Zhang Y., Jia M. (2025). Unveiling the gut microbiota’s role: Monascus fermented ginseng’s impact on hyperlipidemia in vitro. Food Nutr..

[121] Li Z., Kim H.J., Park M.S. (2017). Effects of fermented ginseng root and ginseng berry on obesity and lipid metabolism in mice fed a high-fat diet. J. Ginseng Res..

[122] Park C.H., Kim M., Woo M. (2018). Comparison of the effects of nonfermented and fermented Panax ginseng root against hypertriglyceridemia in high-fat diet-fed mice. J. Med. Food.

